# Simple Sample Preparation Method for Direct Microbial Identification and Susceptibility Testing From Positive Blood Cultures

**DOI:** 10.3389/fmicb.2018.00481

**Published:** 2018-03-20

**Authors:** Hong-wei Pan, Wei Li, Rong-guo Li, Yong Li, Yi Zhang, En-hua Sun

**Affiliations:** ^1^Department of Clinical Laboratory, Qilu Hospital of Shandong University, Jinan, China; ^2^Department of Clinical Laboratory, Jinan Maternal and Child Care Hospital, Jinan, China

**Keywords:** blood cultures, identification, antibiotic susceptibility test, MALDI-TOF MS, Vitek 2 AST systems

## Abstract

Rapid identification and determination of the antibiotic susceptibility profiles of the infectious agents in patients with bloodstream infections are critical steps in choosing an effective targeted antibiotic for treatment. However, there has been minimal effort focused on developing combined methods for the simultaneous direct identification and antibiotic susceptibility determination of bacteria in positive blood cultures. In this study, we constructed a lysis-centrifugation-wash procedure to prepare a bacterial pellet from positive blood cultures, which can be used directly for identification by matrix-assisted laser desorption/ionization-time-of-flight mass spectrometry (MALDI-TOF MS) and antibiotic susceptibility testing by the Vitek 2 system. The method was evaluated using a total of 129 clinical bacteria-positive blood cultures. The whole sample preparation process could be completed in <15 min. The correct rate of direct MALDI-TOF MS identification was 96.49% for gram-negative bacteria and 97.22% for gram-positive bacteria. Vitek 2 antimicrobial susceptibility testing of gram-negative bacteria showed an agreement rate of antimicrobial categories of 96.89% with a minor error, major error, and very major error rate of 2.63, 0.24, and 0.24%, respectively. Category agreement of antimicrobials against gram-positive bacteria was 92.81%, with a minor error, major error, and very major error rate of 4.51, 1.22, and 1.46%, respectively. These results indicated that our direct antibiotic susceptibility analysis method worked well compared to the conventional culture-dependent laboratory method. Overall, this fast, easy, and accurate method can facilitate the direct identification and antibiotic susceptibility testing of bacteria in positive blood cultures.

## Introduction

Bloodstream infection is one of the leading causes of death worldwide (Adhikari et al., 2010; Morgenthaler and Kostrzewa, 2015). Rapid determination of the primary microorganism of infection is crucial for management of a patient with bacteremia (Beekmann et al., 2003; Judd et al., 2014). Matrix-assisted laser desorption/ionization time-of-flight mass spectrometry (MALDI-TOF MS) has proven to be a rapid, accurate, and cost-effective technology in the routine identification of microorganisms (Bizzini and Greub, 2010; Moussaoui et al., 2010; van Belkum et al., 2015; de Almeida et al., 2016), making it possible to now direct identify bacteria from positive blood cultures.

Several recent studies have focused on the development of optimal methods to identify bacteria directly from positive blood cultures using matrix-assisted laser desorption/ionization-time-of-flight mass spectrometry (MALDI-TOF MS). For example, new serum separator tubes (Moussaoui et al., 2010; Stevenson et al., 2010; Wimmer et al., 2012), detergent reagents (Ferroni et al., 2010; Yonetani et al., 2012; Morgenthaler and Kostrzewa, 2015), centrifugation conditions, filters (Fothergill et al., 2013), and commercial kits (La Scola and Raoult, 2009; Juiz et al., 2012; Saffert et al., 2012; Tanner et al., 2017) have been developed to isolate bacteria from positive blood culture samples for direct MALDI-TOF MS identification, allowing for significant reduction of the time to obtaining results, which is now possible within only a few hours (Tian et al., 2016). However, these reported techniques also have some shortcomings such as the requirement of sophisticated equipment, high costs, and relatively low identification accuracy (Morgenthaler and Kostrzewa, 2015; Lin et al., 2017). Furthermore, most of these studies have mainly focused on direct bacterial identification, and there is limited research on methods for the direct determination of the antibiotic susceptibility profiles of the infectious agents identified in positive blood cultures (Romero-Gómez et al., 2012; Croxatto et al., 2014; Barnini et al., 2016; Tian et al., 2016; Bazzi et al., 2017), which is arguably more important for determining appropriate antibiotic treatment than organism identification.

In this study, we aimed to develop a relatively inexpensive and convenient sample preparation method to both directly identify and determine the antibiotic susceptibility profiles of infectious agents in patients with bacteremia. The method was evaluated with clinical bacteria-positive blood samples, and the results were compared with those obtained through conventional laboratory culture-dependent sample preparation procedures (Uki et al., 2013).

## Materials and methods

### Clinical samples

Blood culture bottles (Bactec plus/F; Becton Dickinson, Franklin Lakes, NJ, USA) from patients with a suspected blood infection were collected from September to November of 2017 at Qilu Hospital of Shandong University, Jinan, P.R. China. The blood culture bottles were incubated in the Bactec system (Becton Dickinson) until a positive result was obtained or for a maximum of 5 days. A total of 129 positive blood cultures showing monomicrobial bacterial growth were analyzed using the conventional laboratory diagnostic method and our newly developed method. This study was approved by the ethics committee of Qilu Hospital, Shandong University, Jinan, People's Republic of China [protocol KYLL-2014 (KS)-115]. All subjects provided written informed consent before their inclusion in the study.

### Conventional identification and antibiotic susceptibility testing (AST)

All bacteria-positive bottles were subjected to conventional laboratory MALDI-TOF MS identification and Vitek 2 AST as described previously (Altun et al., 2015; Barnini et al., 2016).

#### MALDI-TOF MS identification

Initially, the positive blood cultures were subjected to Gram staining to determine the presence of gram-positive or gram-negative bacteria. Based on these results, appropriate agar plates, including blood, Maconkey, chocolate, and anaerobic blood agar, were used for further culturing. The plates were grown in an incubator (Thermo Fisher Scientific, USA) at 35°C in a 5% CO_2_ or anaerobic atmosphere until visible colonies appeared. Identification was then performed with the Bruker microflex MALDI-TOF MS system using the MALDI Biotyper 3.0 Realtime classification (RTC) database (Bruker Daltonics, Bremen, Germany) as described previously (Chen et al., 2013; Tian et al., 2016; Zhou et al., 2017). In brief, a pure bacterial colony was smeared onto a steel target plate with a wood toothpick, and 70% formic acid solution was added to lyse the bacterial cells. Once the formic acid solution dried, 1 μL of α-cyano-4-hydroxycinnamic acid (HCCA) matrix (Bruker Daltonics) solution was added for subsequent MALDI-TOF MS (Bruker Daltonics) identification. The calibration and validation of MALDI-TOF MS was carried out once a week with a bacterial test standard (BTS) according to the manufacturer's instructions (Bruker Daltonics).

#### AST

ASTs were carried out with the Vitek 2 system (AES software, bioMérieux, Marcy l'Étoile, France) according to the manufacturer's instructions. Susceptibility cards were inoculated and interpreted according to the Clinical and Laboratory Standards Institute (CLSI) breakpoints (Bazzi et al., 2017). The Vitek cards AST-GN13, AST-Gp67, and AST-Gp68 were used for gram-negative bacteria, staphylococci/enterococci/streptococci (excluding *Streptococcus pneumoniae*), and *S. pneumoniae*, respectively (Tian et al., 2016).

### Workflow for preparation of the bacterial pellet from positive blood cultures

**Figure 1:**
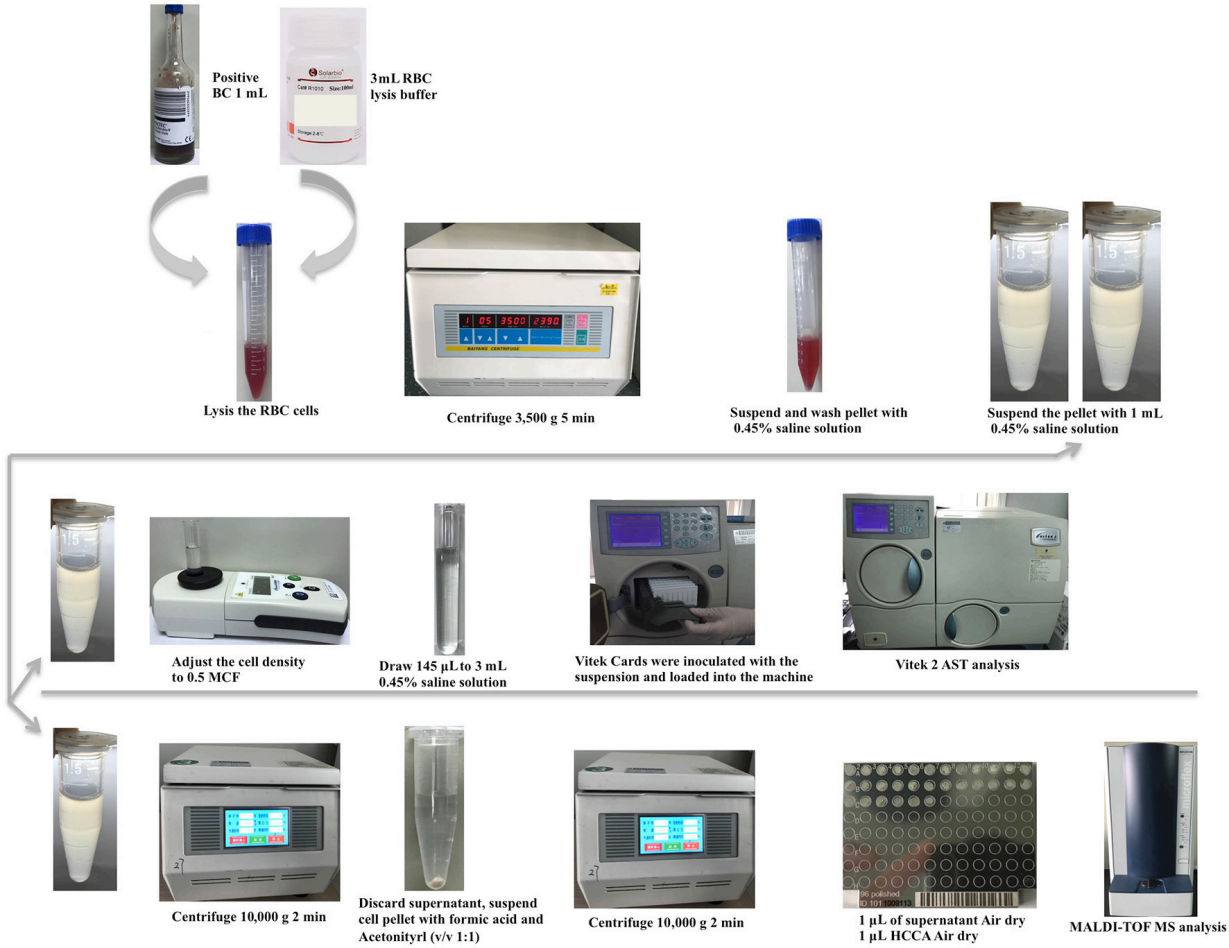
Workflow of the bacterial pellet preparation method for direct identification and antibiotic susceptibility testing.

A key point for the direct identification and AST of microorganisms from positive blood cultures is to effectively isolate and purify bacterial cells from the blood culture medium without affecting cell viability. Ammonium chloride-driven hemolysis has been reported to be a suitable method for red blood cells lysis (Stevenson et al., 2010; Croxatto et al., 2014; Morgenthaler and Kostrzewa, 2015). Therefore, we selected a commercial ammonium chloride buffer solution (Solarbio, Beijing, China; Cat. No. R1010) that can lyse red blood cells without affecting bacteria viability to prepare the bacterial pellet from the positive blood cultures. According to the manufacturer's website, the solution contains 1 g/L KHCO_3_, 8.3 g/L NH_4_Cl, and 0.037 g/L EDTA-Na_2_. We further optimized the experimental protocol and applied a lysis-centrifugation-wash procedure to purify the bacterial pellet from the blood aliquot. The resulting bacterial pellet was then used for direct MALDI-TOF MS identification and Vitek 2 AST as described below (Figure 1).

### Preparation of the bacterial pellet for direct MALDI-TOF MS identification and Vitek 2 AST analysis

A 1-mL aliquot from the positive blood cultures was transferred into a 15-mL micro-centrifuge tube, 3 mL of the lysis buffer (Solarbio) was added, and the final solution was incubated at room temperature until the blood became transparent. The bacterial cells were then pelleted by centrifugation (3,500 × g for 5 min) and re-suspended in 1 mL of 0.45% saline solution to wash the cells. After centrifugation (10,000 × g for 2 min), the supernatant was discarded and the resulting pellet was resuspended in the 0.45% saline solution to adjust the cell density to 3–4 McFarland. The bacterial suspension was then split into two 1.5-mL Eppendorf tubes for direct MALDI-TOF MS (Bruker Daltonics) identification and Vitek 2 (bioMérieux) AST, respectively. The workflow is shown in Figure 1.

#### Direct MALDI-TOF MS identification

One of the above-mentioned bacterial suspensions was pelleted after centrifugation (10,000 × g for 2 min) and the supernatant was discarded. The remaining bacterial cell pellet was dissolved in 50 μL of 70% formic acid thorough vortexing, and 50 μL of pure acetonitrile was added to the solution, vortexed, and centrifuged (10,000 × g for 2 min). One microliter of the supernatant was spotted onto a steel target plate and air-dried, which was immediately overlaid with 1 μL of HCCA matrix solution (Bruker Daltonics) (Chen et al., 2013). Identifications were performed by the Bruker microflex MALDI-TOF MS system using the MALDI Biotyper 3.0 RTC database (Bruker Daltonics) as described above.

#### Direct Vitek 2 AST

Direct Vitek 2 AST was carried out as described above. In brief, the cell density of the remaining bacterial suspension was adjusted to a density of 0.5 McFarland after dilution in 0.45% saline; 145 μL of the bacterial suspension was drawn into 3 mL of 0.45% saline solution to further adjust the bacterial cell density. Vitek cards were inoculated with the suspension vials and loaded into the Vitek 2 automated reader-incubator. Vitek cards AST-GN13, AST-Gp67, and AST-Gp68 were used for gram-negative bacteria, staphylococci/enterococci/streptococci (excluding *S. pneumoniae*), and *S. pneumoniae*, respectively (Tian et al., 2016).

### Data analysis of direct MALDI-TOF MS identification and Vitek 2 AST

Results of the MALDI-TOF MS identification were scored according to the standard criterion of the Bruker microflex MALDI-TOF MS system. In brief, a score ≥2.0 was interpreted as reliable identification to the species level, a score of 1.7–2.0 was interpreted as reliable identification to the genus level, and a score <1.7 was interpreted as no reliable identification. Further experiments verified that scores of 1.6–1.8 were also acceptable for identification to the bacteria species and genus levels (Martinez et al., 2014; Zhou et al., 2017). Based on these criteria, our direct MALDI-TOF MS identification results were compared with those obtained with samples prepared using the conventional laboratory culture-dependent method for evaluation of identification accuracy. Samples determined to be incorrectly identified were those with no peak or a very weak signal, and samples with inconsistent results between the direct identification and conventional identification methods. The correct identification rate was calculated as (correctly identified samples/total tested samples) × 100.

To compare the results of the AST analysis system to those obtained with the existing routine system within a laboratory, CLSI recommends using the metrics of category agreement, minor error, major error, and very major error. Category agreement reflects the number of matches based on the formula (matching categorical results/total tested) × 100. Minor error reflects the degree of susceptible/resistant results versus intermediate susceptibility. Major error indicates a false resistant result, and very major error indicates false susceptibility. Based on these criteria, the minimum inhibitory concentrations obtained by the developed and conventional methods were translated into clinical categories (susceptible, intermediate, and resistant). Our direct Vitek 2 AST results were compared with those of the conventional laboratory culture-dependent samples to evaluate the degree of susceptibility agreement (Tian et al., 2016; Bazzi et al., 2017).

## Results

### Bacterial strains identified

A total of 129 positive blood cultures were analyzed in this study, including 57 gram-negative and 72 gram-positive isolates. The 57 gram-negative isolates included 53 aerobic strains (Table 1) and four anaerobic strains (*Fusobacterium nucleatum, Bacteroides ovatus*, and two *Bacteroides fragilis*). The 72 gram-positive isolates comprised 68 cocci strains and four bacillus strains (Table 1). In addition, *Atopobium (Strep.) parvulum* was identified, which is an anaerobic strain.

**Table 1:** MS Identifiaction results by coventional culture-dependent method and our developed method (*n* = 129).

	**Organisms**	**No. of isolates with score of (*****n*** = **129) culture dependent identification**			**No. of isolates with score of (*****n*** = **129) direct identification**		
		**>2.0**	**1.7–2.0**	**<1.7**	**>2.0**	**1.7–2.0**	**<1.7**
*Gram-negative bacteria* (57)	*Escherichia coli*	20			19	1	
	*Klebsiella pneumoniae*	13		1	14		
	*Pseudomonas aeruginosa*	4	1		4		1
	*Acinetobacter baumannii*		1			1	
	*Enterobacter cloacae*	2			2		
	*Proteus mirabilis*	1			1		
	*Stenotrophomonas maltophilia*	2				2	
	*Burkholderia cepacia*	1			1		
	*Pseudomonas fulva*	1	1		1		1
	*Capnocytophaga sputigena*	1			1		
	*Fusobacterium nucleatum*	1				1	
	*Bacteroides ovatus*	1			1		
	*Chryseobacterium gleum*	2				1	1
	*Bacaeroides fragilis*	2			2		
	*ochrobactrum anthropi*	1					1
	*Leclercia adecarboxylata*	1				1	
*Gram-positive Bacteria (72)*	*Staphylococcus epidermidis*	13	1		2	9	3
	*Staphylococcus aureus*	11			9	2	
	*Staphylococcus capitis*	8	1		6	3	
	*Staphylococcus hominis*	20			16	4	
	*Staphylococcus haemolyticus*	2			1	1	
	*Enterococcus faecium*	5			3	1	1
	*Staphylococcus warneri*		2		1		1
	*Staphylococcus cohnii*			1			1
	*Streptococcus pyogenes*	1				1	
	*Streptococcus sanguinis*	1					1
	*Atopobium (Strep.) parvulum*	1				1	
	*corynebacterium striatum*	1			1		
	*Brevibacterium casei*	1			1		
	*Streptococcus pneumoniae*	1				1	
	*Streptococcus oralis*	1				1	
	*Corynebacterium afermentans*			1			1
Total isolates		119	7	3	86	31	12

### High concordance between direct MALDI-TOF MS and conventional laboratory culture-dependent identification methods

The direct MALDI-TOF MS identification results were compared with those obtained through the conventional laboratory culture-dependent method. Among the 57 samples with gram-negative isolates, 46 (80.70%) showed a score higher than 2, 7 (12.28%) scored between 1.7 and 2, and 4 (7.02%) demonstrated a score below 1.7 (Table 1). Among the 72 samples positive for gram-positive isolates, 40 (55.56%) demonstrated a score higher than 2, 24 (33.33%) demonstrated a score between 1.7 and 2, and 8 (11.11%) demonstrated a score lower than 1.7 (Table 1). Eight of the 12 bacterial isolates with a score lower than 1.7 showed concordant MALDI-TOF MS results with those of the conventional identification. Two gram-negative bacterial strains, *Pseudomonas fulva* and *Ochrobactrum anthropi*, were only correctly identified at the genus level. *Streptococcus oralis* was incorrectly identified as *S. pneumoniae*. In addition, a bacillus strain was unidentified with our direct method, although this strain was identified as *Corynebacterium afermentans* using the culture-dependent method with a score lower than 1.6. Overall, the 57 samples with positive gram-negative isolates exhibited 96.49% (55/57) concordance with the results of conventional laboratory culture-dependent identification, while the 72 samples positive for gram-positive isolates exhibited 97.22% (70/72) concordance with the culture-dependent results.

### Vitek2 direct AST analysis of gram-negative and gram-positive bacteria

Fifty-three of the 57 gram-negative bacterial isolates, including 39 Enterobacteriaceae and 14 non-fermenting gram-negative rods, were selected for direct AST, and the direct AST results from Vitek 2 were compared to those of the conventional laboratory culture-dependent AST method. For Enterobacteriaceae, a total of 658 bacterial-antimicrobial combinations were analyzed. There was a category agreement rate between the two methods of 97.88% of the antimicrobials tested, with 1.82% minor error, no major error, and 0.30% very major error. The minor errors mainly occurred for ampicillin/sulbactam (11.76%), cefepime (5.26%), cefotetan (5.56%), ceftazidime (2.86%), piperacillin-tazobactam (2.63%), tobramycin (2.63%), and ciprofloxacin (2.63%), while the very major errors mainly occurred for sulfamethoxazole/trimethoprim (5.41%). For the non-fermenting gram-negative rods, a total of 177 bacterial-antimicrobial combinations were analyzed. There was category agreement of 93.22% of the antimicrobials tested, with 5.65% minor error, 1.13% major error, and no very major error. The minor errors mainly occurred for meropenem (50%), ciprofloxacin (20%), ceftriaxone (20%), amikacin (10%), and ceftazidime (7.69%), while the major errors mainly occurred for cefepime (10%) and imipenem (10%). The category agreement of all bacterial-antimicrobial combinations is summarized in Table 2.

**Table 2:** Bacterial/antimicrobial combinations and errors in gram-negative bacteria isolates from positive blood cultures.

	**Antimicrobial agent G-b**	**No**.	**Category agreement**		**Minor error**		**Major error**	**Very major error**
Enterobacteriaceae bacteria	Piperacillin-tazobactam	38	37	97.37%	1	2.63%	0	0
	Ceftazidime	35	34	97.14%	1	2.86%	0	0
	Cefepime	38	36	94.74%	2	5.26%	0	0
	Aztreonam	38	38	100%	0		0	0
	Meropenem	23	23	100%	0		0	0
	Imipenem	38	38	100%	0		0	0
	Ertapenem	38	38	100%	0		0	0
	Gentamicin	38	38	100%	0		0	0
	Tobramycin	38	37	97.37%	1	2.63%	0	0
	Amikacin	38	38	100%	0		0	0
	Levofloxacin	38	38	100%	0		0	0
	Ciprofloxacin	38	37	97.37%	1	2.63%	0	0
	Ceftriaxone	38	38	100%	0		0	0
	Sulfamethoxazole/trimethoprim	37	35	94.59%	0		0	25.41%
	Ampicillin/sulbactam	34	30	88.24%	4	11.76%	0	0
	Cefotetan	36	34	94.44%	2	5.56%	0	0
	Ampicillin	37	37	100%	0		0	0
	Cefazolin	38	38	100%	0		0	0
Non-fermentative bacteria	Piperacillin-tazobactam	10	10	100%	0		0	0
	Ceftazidime	13	12	92.31%	1	7.69%	0	0
	Cefepime	10	9	90%	0		1–10%	0
	Aztreonam	10	10	100%	0		0	0
	Meropenem	8	4	50%	4	50%	0	0
	Imipenem	10	9	90%	0		1–10%	0
	Gentamicin	10	10	100%	0		0	0
	Tobramycin	10	10	100%	0		0	0
	Amikacin	10	9	90%	1	10%	0	0
	Levofloxacin	13	13	100%	0		0	0
	Ciprofloxacin	10	8	80%	2	20%	0	0
	Ceftriaxone	10	8	80%	2	20%	0	0
	Sulfamethoxazole/trimethoprim	13	13	100%	0		0	0
	Ampicillin/sulbactam	10	10	100%	0		0	0
	Cefotetan	10	10	100%	0		0	0
	Ampicillin	10	10	100%	0		0	0
	Cefazolin	10	10	100%	0		0	0

Sixty-seven gram-positive bacterial isolates, consisting of 48 coagulase-negative *Staphylococcus* spp., 11 *Staphylococcus aureus*, 5 *Enterococcus* spp., and 3 *Streptococcus* spp. isolates, were used for direct AST analysis. For coagulase-negative *Staphylococcus* spp., a total of 607 bacterial-antimicrobial combinations were analyzed. There was category agreement of 91.27% of the antimicrobials tested, with 5.60% minor error, 1.32% major error, and 1.81% very major error. The minor errors mainly occurred for levofloxacin (23.40%), gentamicin (17.02%), moxifloxacin (14.89%), quinupristin-dalfopristin (6.67%), and ciprofloxacin (6.38%); the major errors mainly occurred for clindamycin (10.64%) and moxifloxacin (4.26%); and the very major errors mainly occurred for clindamycin (6.38%). For *S. aureus*, a total of 143 bacterial-antimicrobial combinations were analyzed. The category agreement was 98.60% for all antimicrobials tested, with 0.70% minor error, no major error, and 0.70% very major error. The errors mainly occurred for erythromycin (minor error, 9.09%) and clindamycin (very major error, 9.09%). For the streptococci isolates, a total of 68 bacterial-antimicrobial combinations were analyzed. The category agreement was 94.12% for all antimicrobials tested, with 2.94% minor error, 2.94% major error, and no very major error. The errors mainly occurred for ciprofloxacin (minor error, 16.67%), tetracycline (minor error, 12.50%), benzylpenicillin (major error, 14.29%), and clindamycin (major error 14.29%). The category agreement of all bacterial-antimicrobial combinations is summarized in Table 3.

**Table 3:** Bacterial/antimicrobial combinations and errors in gram-postive bacteria isolates from positive blood cultures.

	**Antimicrobial agent G+C**	**No**.	**Category agreement**		**Minor error**		**Major error**		**Very major error**	
Coagulase negative staphylococcus	Oxacillin	46	45	97.83%	0		0		1	2.17%
	Benzylpenicillin	47	46	97.87%	0		0		1	2.13%
	Gentamicin	47	38	80.85%	8	17.02%	0		1	2.13%
	Rifampin	46	45	97.83%	1	2.17%	0		0	
	Ciprofloxacin	47	43	91.49%	3	6.38%	0		1	2.13%
	Moxifloxacin	47	37	78.72%	7	14.89%	2	4.26%	1	2.13%
	Levofloxacin	47	36	76.60%	11	23.40%	0		0	
	Clindamycin	47	39	82.98%	0		5	10.64%	3	6.38%
	Erythromycin	47	46	97.87%	0		0		1	2.13%
	Linezolid	47	47	100%	0		0		0	
	Vancomycin	47	47	100%	0		0		0	
	Quinupristin-dalfopristin	45	42	93.33%	3	6.67%	0		0	
	Tetracycline	47	43	91.48%	1	2.13%	1	2.13%	2	4.26%
*Staphylococcus aureus*	Oxacillin	11	11	100%	0		0		0	
	Benzylpenicillin	11	11	100%	0		0		0	
	Gentamicin	11	11	100%	0		0		0	
	Rifampin	11	11	100%	0		0		0	
	Ciprofloxacin	11	11	100%	0		0		0	
	Moxifloxacin	11	11	100%	0		0		0	
	Levofloxacin	11	11	100%	0		0		0	
	Clindamycin	11	10	90.91%	0		0		1	9.09%
	Erythromycin	11	10	90.91%	1	9.09%	0		0	
	Linezolid	11	11	100%	0		0		0	
	Vancomycin	11	11	100%	0		0		0	
	Quinupristin-dalfopristin	11	11	100%	0		0		0	
	Tetracycline	11	11	100%	0		0		0	
*Enterococcus* spp. and *Strepcoccus* spp.	Benzylpenicillin	7	6	85.71%	0		1	14.29%	0	
	Ciprofloxacin	6	5	83.33%	1	16.67%	0		0	
	Moxifloxacin	8	8	100%	0		0		0	
	Levofloxacin	8	8	100%	0		0		0	
	Clindamycin	7	6	85.71%	0		1	14.29%	0	
	Erythromycin	8	8	100%	0		0		0	
	Linezolid	8	8	100%	0		0		0	
	Vancomycin	8	8	100%	0		0		0	
	Tetracycline	8	7	87.50%	1	12.50%	0		0	

## Discussion

Immediate administration of appropriate antibiotics is necessary for the effective treatment of bacteremia, as any delay is associated with increased morbidity and mortality (Ascione et al., 2015). Several studies have demonstrated the successful application of MALDI-TOF MS in direct identification of bacteria from positive blood cultures (Schneiderhan et al., 2013; Morgenthaler and Kostrzewa, 2015). However, the major limitations of the current technologies are that they are time-consuming, expensive, complicated, and show relatively low identification accuracy. Moreover, most of these previous studies focused on direct bacterial identification, with little application of a direct culture-independent AST method (Lupetti et al., 2010; Barreales et al., 2011; Croxatto et al., 2014; Jo et al., 2016; Tian et al., 2016). Thus, an accurate, rapid, and cheaper combined method for direct identification and AST of the infectious agents from positive blood cultures is still in demand.

Using an iron-based buffer solution, we successfully developed a simple lysis-centrifugation-wash procedure (Figure 1) to prepare bacterial pellets from positive blood cultures for application in direct identification and AST. This sample preparation process took <15 min to complete, which is much faster than the conventional laboratory biological procedures as well as the majority of other reported protocols. The main advantage of our method is that the prepared bacterial pellet can be used for both direct MALDI-TOF MS identification and direct AST, making it even more efficient. Moreover, the proposed protocol is relatively simple for application by clinical laboratory technicians. The cost of our method is estimated at <$0.35 per sample, which is cheaper than the commercial Sepsityper™ kit ($7/sample) or other reported sample preparation methods for MALDI-TOF MS (Zhou et al., 2017).

Overall, we determined a correct rate of the direct identification of over 96% using our proposed method, suggesting that our method has relatively improved performance compared to those reported previously. Consistent with other studies (Prod'hom et al., 2010), no difference was observed in the identifications of isolates, with most spectral scores ranging between 1.7 and 2 or higher than 2. Although six gram-positive bacteria and two gram-negative bacteria showed identification scores lower than 1.7, the identification results were largely consistent with those of the culture-dependent method. In particular, several slow-growing and rare bacterial strains (such as *Capnocytophaga sputigena* and *A. parvulum*) could also be correctly identified by our method. The accurate and quick identification of such bacterial strains is meaningful for making appropriate clinical treatment decisions. However, our method misidentified *S. oralis* as *S. pneumoniae*, which may reflect the limitation of MALDI-TOF MS, as previously reported (Prod'hom et al., 2010). Low identification scores (<2.0) were mainly associated with coagulase-negative staphylococci such as *Staphylococcus epidermidis, Staphylococcus hominis*, and *Staphylococcus capitis* that are commonly recognized as blood culture contaminants. Moreover, our results indicated that most samples with lower bacterial density usually demonstrated identification scores lower than 1.7. It is probably because of the insufficient biomass that could not produce clear MALDI-TOF MS signals.

The overall category agreement of the direct antimicrobials tested was over 90%, with the highest rates of concordance observed for Enterobacteriaceae and *S. aureus*, in line with the results of previous studies (Wimmer et al., 2012; Tian et al., 2016). According to the CLSI interpretive criteria (Bazzi et al., 2017), the overall error rate of our method is acceptable. Moreover, the error rate of gram-negative bacteria was relatively lower than that of gram-positive bacteria, which is consistent with other studies.

For Enterobacteriaceae, the categorical agreement was >90% for most of the antibiotics tested, with the exception of only ampicillin/sulbactam (88.24%), whereas relatively lower categorical agreement rates were detected for meropenem, ciprofloxacin, and ceftriaxone for non-fermenters. The error in meropenem was mainly detected for *Pseudomonas* spp., suggesting that the direct meropenem AST is not applicable for this genus. Nevertheless, 100% categorical agreement was detected for meropenem, imipenem, and ertapenem against Enterobacteriaceae, indicating that our method is highly applicable for detection of carbapenem resistance in Enterobacteriaceae.

With respect to the gram-positive bacteria, AST disagreement was mainly observed in coagulase-negative staphylococci, including gentamicin, moxifloxacin, levofloxacin, and clindamycin. Coagulase-negative *Staphylococcus* strains are usually recognized as contamination species in blood samples. Indeed, when excluding these strains, our method showed greater accuracy. The category agreement of direct antimicrobials tested against *S. aureus, Enterococcus* spp., and *Streptococcus* spp. reached up to 97.20%. There was only one minor error (erythromycin) and one very major error (clindamycin) in the direct AST results of *S. aureus*, and this species showed 100% categorical agreement of oxacillin, demonstrating that our method is suitable for detection of methicillin-resistant *S. aureus* (MRSA). Although only five enterococci isolates were tested in this study, 100% categorical agreement was observed for these isolates with linezolid and vancomycin, suggesting that our method is probably also effective for the detection of vancomycin-resistant enterococci (VRE); however, more enterococci samples are needed for further verification of these AST results.

In summary, we have developed an easy, fast, and accurate combined method for the direct identification and AST analysis of bacteria in positive blood cultures. The direct Vitek 2 AST worked particularly well for the detection of carbapenem-resistant enterococci, MRSA, and VRE isolates. Timely detection of those isolates can further alert clinicians to adjust the treatment or initiate essential combination therapy, which will significantly reduce mortality, morbidity, and hospital costs. One important limitation of our study is the relatively low number of samples analyzed, especially for non-fermenters and enterococci. Therefore, in subsequent studies, we plan to include more of such cases to further evaluate our method. Moreover, the current method does not work well for yeast isolates and polymicrobial blood cultures (data not shown). Therefore, efforts are currently under way to optimize the method for yeast identification and AST in positive blood cultures.

## Author contributions

YZ, HP, ES, and RL conceived and designed the experiments; HP, ES, WL, and YL performed the experiments; HP and ES wrote the paper. All authors reviewed the manuscript.

### Conflict of interest statement

The authors declare that the research was conducted in the absence of any commercial or financial relationships that could be construed as a potential conflict of interest.
